# Underwater visibility constrains the foraging behaviour of a diving pelagic seabird

**DOI:** 10.1098/rspb.2022.0862

**Published:** 2022-07-13

**Authors:** J. Darby, M. Clairbaux, A. Bennison, J. L. Quinn, M. J. Jessopp

**Affiliations:** ^1^ School of Biological, Environmental and Earth Sciences, University College Cork, Cork T23 N73K, Ireland; ^2^ MaREI Centre for Energy, Climate and Marine, Environmental Research Institute, University College Cork, Cork P43 C573, Ireland; ^3^ British Antarctic Survey, Madingley Road, Cambridge CB3 0ET, UK

**Keywords:** climate change, Manx shearwaters, prey detection, seabird foraging, sensory ecology, turbidity

## Abstract

Understanding the sensory ecology of species is vital if we are to predict how they will function in a changing environment. Visual cues are fundamentally important for many predators when detecting and capturing prey. However, many marine areas have become more turbid through processes influenced by climate change, potentially affecting the ability of marine predators to detect prey. We performed the first study that directly relates a pelagic seabird species's foraging behaviour to oceanic turbidity. We collected biologging data from 79 foraging trips and 5472 dives of a visually dependent, pursuit-diving seabird, the Manx shearwater (*Puffinus puffinus*). Foraging behaviour was modelled against environmental variables affecting underwater visibility, including water turbidity, cloud cover and solar angle. Shearwaters were more likely to initiate area-restricted search and foraging dives in clearer waters. Underwater visibility also strongly predicted dive rate and depth, suggesting that fine-scale prey capture was constrained by the detectability of prey underwater. Our novel use of dynamic descriptors of underwater visibility suggests that visual cues are vital for underwater foraging. Our data indicate that climate change could negatively impact seabird populations by making prey more difficult to detect, compounded by the widely reported effects of reduced prey populations.

## 1. Introduction

The chemical and physical properties of the planet's oceans are changing at an unnatural rate [1], bringing about challenges for marine life. A changing climate is exaggerating the physical processes that increase ocean turbidity, such as wave action and seabed shear stress. These forces accelerate the rate of sediment resuspension in productive shelf sea regions [2], leading to lower light transmissibility through seawater over broad spatio-temporal scales [3–5]. This suspension of non-algal particulate matter can negatively affect primary producers [6], potentially impacting the base of marine food webs. Climate-driven physical and chemical changes, including warming, stratification and carbon enrichment of seawater, are also affecting the timing and intensity of plankton blooms, altering the light and nutrient availability of oceanic habitat, often over vast areas [7].

An increasingly turbid ocean may have negative consequences for oceanic consumers that use visual cues for prey capture. Some fishes have reduced movement efficiency when foraging in turbid conditions [8,9], and one study found that fish biomass in the North Sea was 76–85% positively correlated with water visibility alone [10], suggesting that visibility is pivotal in the habitat preference of many fish species. In addition, species compositions can be altered by elevated turbidity in coastal systems, where visual predators are put at a disadvantage compared to chemosensory predators [11]. The effect of such visibility/turbidity on prey detection and foraging has been widely explored in freshwater [12,13] and estuarine systems [14], where turbidity levels are often higher, but where prey capture and foraging usually occur over small scales. While many marine species have been shown to rely on light levels and visual cues for foraging [15–18], the effect of turbidity and reduced visibility on foraging efficiency has received little attention in oceanic systems.

Seabirds rely on a sensory array for prey detection over an expansive and seemingly featureless ocean. Chemoreception and olfaction are thought to influence the broad-scale search behaviour of tubenose seabirds (order: *Procellariiformes*), with indicator compounds (e.g. dimethyl sulfide, pyrazines) likely to attract these birds towards areas of high productivity and prey availability [19,20], or to fishing vessels beyond the range of visual detection [21–23]. Great cormorants (*Phalacrocorax carbo*) have been shown to use acoustic cues underwater for prey capture in highly turbid coastal regions [24] where low-visibility may benefit non-visual methods of prey detection. Acoustic communication between conspecifics has also been observed during gregarious feeding in foraging Cape gannets [25], and three penguin species [26], though it is unclear whether hearing is involved in prey detection and capture in these species.

For many seabirds, visual cues are probably essential for foraging. Cameras attached to Scopoli's shearwaters (*Calonectris diomedea*) showed that individuals targeted aggregations of conspecifics, often in association with other marine predators, before engaging in foraging behaviour [27]. Bird-borne cameras on Cape gannets (*Morus capensis*) led one study to conclude that broad scale search for food mostly relied on stimuli visible above the water, such as fishing vessels, conspecifics or other predators [28]. On a finer scale, gannet species are also thought to use sight to locate conspecifics before diving, either to avoid collisions or to attempt to steal their prey [29]. The importance of underwater vision for prey capture by seabirds is more difficult to quantify [30], though some studies would suggest that foraging capabilities of diving seabirds is limited by underwater visibility [31]. A study on penguin species suggested that diel patterns in maximum dive depth were dependent on light availability due to solar angle, rather than on the vertical distribution of prey [15]. Captive little penguins (*Eudyptula minor*) were shown to reduce prey capture attempts with decreasing light availability [32]. Hundreds of thousands of short-tailed shearwaters (*Puffinus tenuirostris*) starved to death in 1997 during an anomalous coccolithophore bloom in their Bering Sea wintering grounds that drastically reduced light transmission through the water [33]. The shearwaters' preferred prey shifted their vertical distribution towards deeper waters, probably to avoid anomalous surface temperatures. This reduced prey availability may have been compounded by the widespread increases in turbidity, further impairing their ability to detect prey [34]. One study suggests that these die-offs occurred due to difficulty in visually detecting prey from above water, though this study was based on modelled prey capture strategies rather than empirical measurements of foraging effort or dive depth [35].

Understanding the sensory ecology of a species is vital if we are to predict how sensitive it is to changes in its environment. Increasingly turbid oceans due to climate change are likely to constrain the foraging abilities of visual pursuit hunters, with knock-on effects on annual survival and reproductive output. The Manx shearwater (*Puffinus puffinus*) is an ideal model species for investigating the effects of turbidity on foraging; they are highly mobile, undertaking foraging trips up to thousands of kilometres from their colony [36,37] ranging over an area of continental shelf that has become increasingly turbid in recent decades [3]. While Manx shearwaters probably use olfactory cues for broad-scale search behaviour [20], the physiology and placement of their eyes indicate that prey capture relies on visual guidance [38]. Manx shearwaters actively pursue prey underwater at depths down to 50 m, with dives limited to daylight hours, suggesting that light availability is important for the pursuit of prey [39]. Here we investigated the role of variables that determine underwater visibility including solar angle, cloud cover and turbidity on the broad-scale search patterns, dive rate and maximum dive depth of foraging Manx shearwaters.

## 2. Methods

### (a) Data collection

A total of 36 breeding adult Manx shearwaters were successfully tracked from Little Saltee (52.138, −6.586), Ireland, from June to August 2021. All capture, handling and tagging was completed under licence from the National Parks and Wildlife Service (54/2021, C155/2021) and the British Trust for Ornithology (CO/6143). Birds were either caught by hand using nest access chambers, or purse nets at the nest entrance [40]. Pathtrack nanoFix Geo (3.5 g) with integrated time-depth recorder (TDR, *n* = 14) or CatLog genII+ (approx. 10.5 g) tags (*n* = 32) were attached to feathers on the centre of the bird's back using Tesa 4651 waterproof tape. Both tag types were set to record high accuracy GPS fixes at 5-minute intervals. CatLog GPS tags were paired with Cefas G5 TDRs (2.5 g) on 8 individuals. PathTrack TDRs recorded depth at 0.5 Hz when underwater, and had an accuracy of ±1% up to 50 m and a resolution of 1 cm. Cefas TDRs were set to record depth at 0.5 Hz constantly, and 4 Hz when underwater, and had an accuracy of ±1% and a resolution of less than 4 cm. All depth data were subsampled to 0.5 Hz to match the temporal resolution of the PathTrack TDRs. Total weight of devices and attaching material were 3% or less of the bird's total mass (mean ± s.d. = 2.5 ± 0.1%). Tags were mounted on the bird's back, slightly behind the highest point, to mitigate against negative aerodynamic and hydrodynamic impacts of tag attachment [41,42].

### (b) Foraging trips and dive locations

All analyses were completed using R v. 4.1.2 (cran.r-project.org). Tracks were linearly interpolated to consistent 5-minute intervals using *PathInterpolatR* (github.com/jedalong/PathInterpolatR) to correct for any delayed or missing GPS fixes (typically when a bird was underwater at the time of the location fix attempt). Where gaps of greater than 1 h were present in the raw GPS data, tracks were split into sections to avoid interpolating over large time intervals. Foraging trips were defined as when an individual spent at least 6 h greater than 5 km from the colony, with track points at the colony (1 km radius) removed from further analysis [43]. Concurrent GPS and TDR data were recorded for 15 individuals across 29 foraging trips. Dives were identified as a sequence of consecutive depth data for which depth was greater than 1 m. Dives were further grouped into bouts of diving activity, split by time intervals between dives, and the bout ending criterion defined with nonlinear least-squares regression using the *diveMove* package (cran.r-project.org/web/packages/diveMove). Locations were appended to dives from the closest track point timewise, and similarly number of dives was calculated for each track point interval.

### (c) Environmental variables

Environmental variables that directly measure or affect water visibility were appended to track and dive data by date, time, and location. Solar angle (**°**) was calculated using the *oce* package (cran.r-project.org/web/packages/oce/) and used as a proxy for light availability. Solar angle was taken as the angle between the sun and horizon, with positive values above the horizon, negative values below, and 0 at rising or setting. Secchi disc depth (Zsd) was used as a metric of light transmissibility through the water column, i.e. turbidity [10,44]. Zsd was provided in metres, where greater Zsd corresponds to clearer water, and sourced at daily temporal and 4 km spatial resolution from the Copernicus Marine Service Ocean Products database (resources.marine.copernicus.eu/). Cloud cover (%) data were sourced from MoveBank's Env-DATA service (www.movebank.org/), which accesses the European Centre for Medium-range Weather Forecasts' ERA5 dataset [45]. These data are provided at 0.25 × 0.25 degrees spatial and hourly temporal resolution and were appended using bilinear interpolation. Water depth (m) was calculated using the *marmap* package at 2 arc-minute resolution (cran.r-project.org/web/packages/marmap). Time of day was calculated as hours from midnight in Universal Time Zone.

### (d) Informing hidden Markov models using Secchi disc depth

We investigated whether water Zsd could improve model fit for a behavioural classification method currently used for marine top predators. Hidden Markov models (HMMs) can be used to distinguish between three putative behavioural states using step length and turning angle in seabird tracking data: rest, area-restricted search (ARS) and transit [46,47]. ARS is thought to represent the movement mode most likely to include prey capture attempts, usually with steep turning angles and intermediate distances between points [48]. Environmental variables that may affect the decision of an animal to engage in one of these behaviours can be included in these models to improve fit [49]. Initial values for these parameters were taken from a previous study [20], who fit HMMs using Manx shearwater tracks at the same temporal resolution from colonies on the west coast of Ireland. Two HMMs were run using the *MomentuHMM* package (cran.r-project.org/web/packages/momentuHMM/), one with and one without Zsd as a model covariate. The AIC of these models were compared to assess how Zsd affected model fit. For tracks with concurrent TDR data, the proportion of dives within each state of each HMM (with and without turbidity as a covariate) was also calculated to assess behavioural prediction accuracy, comparing hit rate, miss rate and precision across models. Stationary state and state-switching probabilities were calculated as a response to Zsd using the *plotStationary* function in *momentuHMM*.

### (e) Modelling dive rate

Dive rate was modelled using a generalized additive mixed-effects model (GAMM) with bird identity included as a random effect to account for variation caused by tagging effects and/or individual differences in target prey or maximum dive depth. We used the *bam* function in the *mgcv* package (cran.r-project.org/web/packages/mgcv/) which allows for the efficient fitting of generalized additive models (GAMs) with an autoregressive order 1 (AR(1)) structure. An autocorrelation function (ACF) was used to establish a coefficient (*rho*) to describe serial correlation between track points. Thin-plate regression splines with shrinkage were used for all predictor variables, which return the simplest effective spline. The model gamma parameter was set to 1.2, which increases the null-space penalty to avoid overfitting of model terms [50]. Model selection was performed using an inbuilt feature in *mgcv*'s model fitting infrastructure, which uses spline shrinkage to regress a covariate's effect to 0 where it has no significant effect on the model response. The response variable, dive rate, was presented as dive count per 5 min using a negative binomial model structure with a log link to account for overdispersion. Solar angle, cloud cover and Zsd were included as explanatory variables, as all will affect water visibility. Time of day was also included to account for diel patterns in dive rate not attributable to light levels and was fit using a cyclic cubic spline. A two-dimensional spline was chosen to represent solar angle and Zsd, as both work in combination to regulate light transmission through water. A tensor product spline was used for this two-dimensional relationship because of the differing scales of these two variables. This method was validated by comparing Akaike's information criterion (AIC) values for two models, one with the two-dimensional spline and one with two individual splines. Model goodness of fit (GOF) was described using deviance explained. Area under the receiver operating characteristic curve (AUC) was also calculated as a secondary measure of GOF using the *caret* package (cran.r-project.org/web/packages/caret/). We predicted a binomial response using the fitted model and compared the prediction to the presence or absence of dive behaviour for each track location as a Boolean object to calculate AUC.

### (f) Modelling dive depth

Maximum dive depth was also modelled as a response to environmental covariates using a GAMM. The model response was maximum depth per dive bout (*n* = 1358), to account for fine-scale variation in dive depths within bouts of diving behaviour. Bird identity was again included as a random effect. No serial correlation was observed in the ACF plot of this model's residuals, so no autocorrelation structure was implemented. A Gaussian error structure with an identity link was used based on the distribution of model residuals. Zsd, solar angle, and cloud cover were included, considering these variables regulate light levels, which are likely to influence dive depth [15]. Zsd and solar angle were again tested as both a two-dimensional tensor product spline and two individual splines using AIC to select the better descriptor. Time of day was included to capture any changes in dive depth based on vertical distribution of prey and how that may change throughout the day irrespective of light levels [18]. Water column depth was also included, as this forms a physical constraint to maximum dive depth that needs to be considered. HMM inferred state was included as a covariate to compare dive depths across different phases of motion, represented as transit, ARS and resting on the water. A second model was also run with the tensor product of solar angle and Zsd fit to each study individual separately, and these effects were then compared superficially to the same tensor product in the overall model.

## 3. Results

### (a) Foraging trips and dive locations

**Figure 1.**
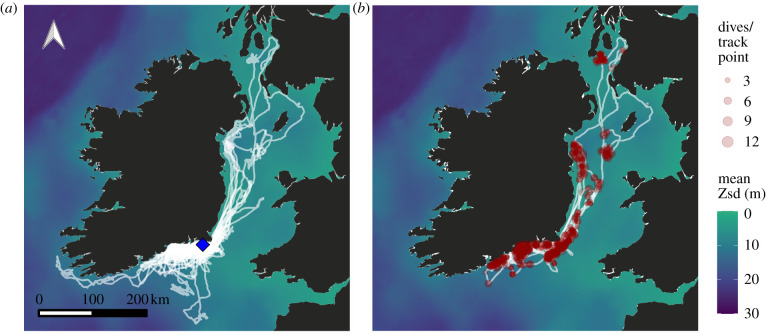
(*a*) Manx shearwater foraging trips (*n* = 79) from Little Saltee. Colony shown by the blue point. (*b*) Recorded dives of Manx shearwaters. Each point represents a track location with dives associated with it. The size of the point corresponds to the number of dives per track location, and all are 30% opaque to better visualise spatial overlap. Only trips with associated dive data (*n* = 30) are retained in this map. The background shows the mean Zsd (m) across the study period. This mean is only used for visualization, and dynamic daily values were instead used for all analysis. (Online version in colour.)

A total of 79 foraging trips were recorded from 36 breeding Manx shearwaters on Little Saltee. 5472 individual dives were recorded from the 15 study individuals also equipped with TDRs, with a mean ± s.d. of 67 ± 33 dives per day (range 5–134). Mean ± s.d. dive depth was 8 m ± 6.5, ranging up to 42 m. Mean ± s.d. Zsd encountered on each foraging trip was 7.5 m ± 2.6. Tracks were mostly distributed along the south and east coasts of Ireland (figure 1). Dives almost all occurred during daylight hours, with peaks of occurrence around dawn and dusk (electronic supplementary material, figure S1).

### (b) Informing hidden Markov models using turbidity

**Figure 2.**
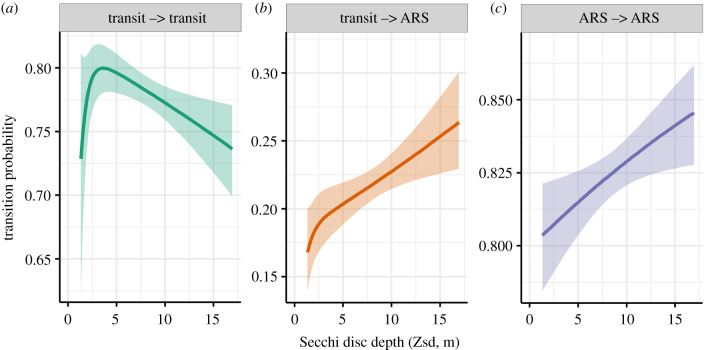
Transition probabilities between behavioural states affected by Secchi disc depth (m) according to the three-state HMM. Shaded areas represent 95% confidence intervals. See electronic supplementary material, figure S3 (supporting information) for a full matrix of transition probabilities in relation to Secchi disc depth. (Online version in colour.)

The fit of the three-state HMM was improved by including Zsd as a covariate according to AIC. The states assigned by each model (with and without Zsd) were 99.5% similar. Model prediction hit rate stayed the same with the inclusion of Zsd, though miss rate and precision were both negatively affected, suggesting that Zsd improved model fit based on movement phases alone, but did not improve the prediction of diving behaviour (electronic supplementary material, table S1). Increasing Zsd led to a higher likelihood of switching from transit to ARS states, and individuals were also more likely to remain in an ARS state when Zsd was higher (figure 2). Of the track points that contained dives, 75.5% occurred in an inferred ARS state (electronic supplementary material, table S2), and the stationary probabilities indicate that ARS behaviour was more likely to occur in areas of high Zsd (electronic supplementary material, figure S2).

### (c) Modelling dive rate

**Figure 3.**
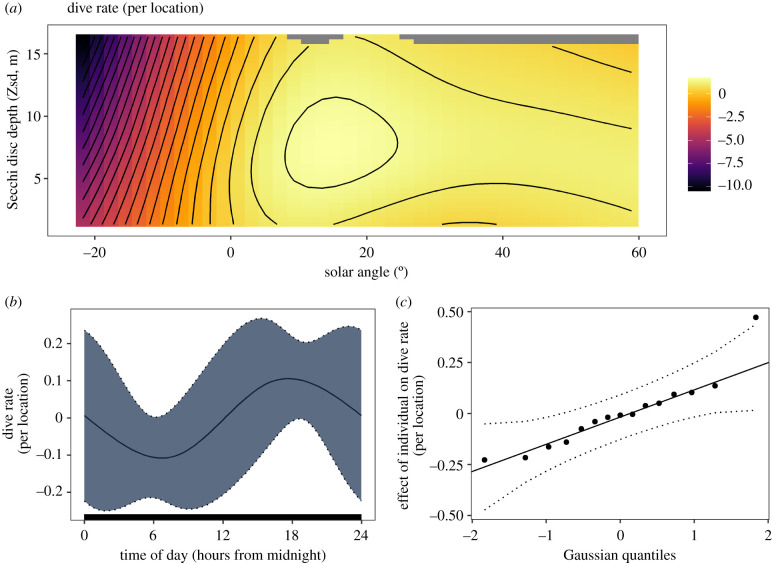
Significant GAMM covariates describing the dive rate of Manx shearwaters. (*a*) For the two-dimensional effect of Secchi disc depth (Zsd) and solar angle, the fill colour represents covariate effect on dive rate. A log link function was used to fit the negative binomial distribution, so true effect on dive rate is calculated as the exponential of the displayed effect. (*b*) The effect of time of day. The *y*-axis represents the effect on dive rate. (*c*) The range of effects that individual ID has on dive rate, with each plot point representing an individual. The 95% confidence interval of time of day and individual ID model terms are delineated by dotted lines. (Online version in colour.)

Dive rate was predicted by the two-dimensional tensor product of solar angle and Zsd, as well as time of day and individual ID. Cloud cover did not have a significant effect (table 1). The effect of solar angle and Zsd on dive rate clearly reflects a diurnal pattern of diving behaviour, with a peak around lower positive values of solar angle corresponding to dawn and dusk (figure 3*a*). Moderate Zsd led to higher dive rates, particularly in the dawn/dusk peak (figure 3*a*). Time of day suggests that dive rate increases during the evening, with higher rates in the latter half of the day (figure 3*b*). The significant effect of individual identity indicates between individual variation in dive rate (figure 3*c*). This model explained 22% of deviance in dive rate, while the AUC for dive prediction was 74%, signifying moderate to good model GOF.

**Table 1.** Dive rate GAMM covariates. Response is dive count per location at 5-minute intervals. Estimated degrees of freedom (EDF) is a measurement of term complexity, *F*-statistic represents effect on the model output, and terms with a *p*-value less than 0.05 are taken to be significant (bold text, * symbol after *p*-value).

model covariate	EDF	*F*-statistic	*p*-value
**tensor product (solar angle × Zsd)**	**12.4**	**6.9**	<0.001*
**time of day**	**0.8**	**1.9**	0.027*
**ID (random)**	**7.5**	**1.4**	<0.001*
cloud cover	<0.01	0	0.77

### (d) Modelling dive depth using water turbidity

**Figure 4.**
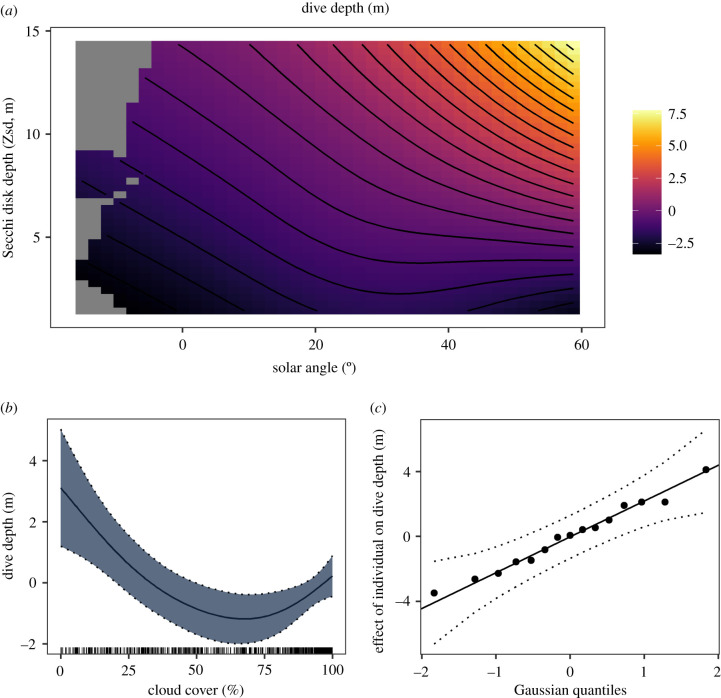
Model covariates describing the dive depth of Manx shearwaters. (*a*) For the two-dimensional effect of Secchi disk depth (Zsd) and solar angle, the fill colour represents covariate effect on dive depth. (*b*) One-dimensional effect of cloud cover. The *y*-axis represents the covariates' effect on dive depth, and the rug plot beneath reflects the distribution of values. (*c*) The range of effects that individual ID has on dive rate , with each plot point representing an individual. The 95% confidence interval of the effect of one-dimensional and random model terms are delineated by dotted lines. (Online version in colour.)

The tensor product of solar angle and Zsd, as well as cloud cover and individual ID predicted maximum dive depth per bout of diving behaviour (table 2). When this effect was tested on a per-individual basis, a similar effect was observed where the individual tensor product was significant (electronic supplementary material, figure S5), which suggests that the relationship is robust and consistent across individuals. Water depth was selected against, as this model term was regressed to 0 by the selection process and had no detectable effect on maximum dive depth per bout. High solar angle and Zsd together led to greater dive depths, suggesting that maximum dive depth is constrained by light levels available underwater (figure 4*a*). This is reinforced by the lack of diving at night, with less than 1% of dives occurring after civil twilight (solar angle <−6 degrees) throughout the entire dataset (electronic supplementary material, figure S1). Cloud cover had a negative effect on dive depth overall, though the relationship was not fully linear, with dive depth increasing slightly between moderate and high total cloud cover (figure 4*b*). Time of day did not have a significant effect, but the term was retained by the model selection process and had a non-zero effect (table 2), indicating that the dive depth may increase later in the day, as was observed for dive rate (figure 3*b*), though the effect is weak. The random effect of individual identity was also significant, probably due to variation in individual fitness, tagging effects, and/or depth of preferred prey (table 2 and figure 4*c*). The deviance explained by this model was 12.5%, increasing to 15% when the tensor product of solar angle and Zsd was split according to individual.

**Table 2.** Maximum dive depth GAMM covariates included as smooth terms. Response is maximum dive depth per bout of diving behaviour (m). Estimated degrees of freedom (EDF) is a measurement of term complexity, *F*-statistic represents effect on the model output, and terms with a *p*-value less than 0.05 are taken to be significant (bold text, * symbol after *p*-value).

model covariate	EDF	*F*-statistic	*p*-value
**tensor product (Solar angle × Zsd)**	**3**.**8**	**2**.**4**	**<0**.001*
**cloud cover**	**2**.**2**	**4**.**4**	**0**.**002**
**ID (Random)**	**10**.**5**	**3**.**8**	**<0**.001*
time of day	0.3	0.3	0.18
depth	<0.01	0	0.59

Behaviour inferred by the HMM had a significant effect on dive depth (electronic supplementary material, table S3, figure S4). There was no significant difference between dive depths in inferred rest or ARS states, but dives were 2.9 m shallower when they occurred in inferred transit states (*p*-value = 0.004).

## 4. Discussion

Using detailed spatio-temporal analysis, we demonstrated that Manx shearwater foraging behaviour is affected by water visibility. At fine scales, high solar angles, clear waters and low cloud cover all lead to greater maximum dive depths. Both dive depth and rate were best explained when solar angle and turbidity were combined into a single two-dimensional covariate, which strongly infers that diel dive patterns observed are limited by light availability. Less than 1% of dives occurred when the sun was more than 6 degrees below the horizon. This also suggests that dives were preceded by visual detection of either prey or indicators of prey, such as other predators [27,51]. Cloud cover had no effect and turbidity had a minimal effect on dive rate, reinforcing the hypothesis that visual stimuli for dives probably occur at or close to the water surface [28]. Dive rate decreased slightly at very low turbidity levels, though this may simply reflect deeper, longer and more energetically costly dives undertaken due to elevated visibility, resulting in a reduced capacity for dives within the 5-minute window. Dive rate increased before dusk, which may have coincided with an increased availability of prey, or increased foraging effort prior to returning to the colony to provision their chick at night. This late peak in diving behaviour is not limited to the final day of each foraging trip before returning to the colony (electronic supplementary material, figure S1, supporting information), and is most likely to be driven by temporal increases in prey availability. Nonetheless, further data on foraging success during dives, e.g. from bird-borne cameras [27], are necessary to investigate this temporal trend.

The three-state hidden Markov model fit was improved by including Secchi disc depth as a measure of turbidity. Switching from transit to ARS was more likely over low turbidity waters, as was remaining in ARS. This model also inferred a slightly higher stationary probability of ARS in areas of low turbidity, meaning that movement patterns consistent with broad-scale search behaviour and more likely in clearer waters overall. These models are not infallible predictors of behaviour [46], and 24.5% of track points with dives were not within the inferred ARS state. Dives during inferred rest behaviour may reflect periods of preening and maintaining feathers between dives over good quality habitat which will confound behavioural classification based on two-dimensional GPS track data alone. Dives that occurred while the bird was inferred to be in transit between prey patches were significantly shallower than during other modes of movement, consistent with opportunistic visual detection of prey or prey indicators during directed flight [52,53].

Cloud cover had a mostly negative effect on dive depth. Clear skies led to the greatest maximum depths, which is intuitive when light availability is taken to be a limiting factor [15]. High cloud cover also led to slightly greater dive depths than intermediate cloud cover, suggesting that complete cloud cover may covary with prey availability at certain depths. This could occur through mixing at ocean front systems for instance [54], as sea-surface temperature gradients at frontal mixing zones can create dense cloud cover through accelerated atmospheric convection [55]. Manx shearwaters possess either violet sensitive (VS) or ultraviolet sensitive vision (UVS) [56,57]. Clouds don't attenuate these shorter wavelengths of light to the same degree as longer wavelengths in the visible spectrum [58], so complete cloud cover may not limit availability of Manx shearwater's visible spectra as much as we might expect. However, violet and ultraviolet light are attenuated at a much greater rate in turbid waters compared to other visible wavelengths [59,60]. Therefore, while VS or UVS vision may confer an advantage in heavy cloud cover, it might also be impaired to a greater degree in turbid water, giving further context to the restrictive effect of turbidity on dive depths in Manx shearwaters.

The relatively coarse spatial and temporal resolution of environmental variables may account for some of the unexplained variation in our models, along with other unknown factors, the most obvious of which are vertical prey distribution and varying effects of device attachment. Despite this, the results described here are biologically logical. Manx shearwaters possibly also capture prey at the water surface, which we can't confidently identify using the existing data streams. This behaviour might be identified using additional data streams, such as from accelerometers [61]. While quantifying surface prey capture may provide additional insight into the foraging ecology of Manx shearwaters, the findings of this study pivot specifically around their diving behaviour, so this potential knowledge gap is not critical to our conclusions.

Turbidity limiting the foraging ability of marine visual predators has wide-ranging conservation implications. Large marine areas have become more turbid in recent decades, driven by increased wave action and seabed shear stress associated with climate change [2,3]. This affects both shallow coastal and deeper offshore shelf waters [4,5]. Such a widespread decrease in light transmissibility through water is certain to have a negative effect on visual foragers occupying many trophic levels in these areas [10,62], as well as reducing light availability for primary producers [5]. Extreme storms events, which are already becoming more frequent in areas such as the North Atlantic [1] and forecast to increase in frequency in some of the most biodiverse oceanic areas [63,64], may also acutely reduce visibility. Such storms are responsible for mass mortality events in seabirds, especially those with reduced mobility due to high wing loading or flight feather moult, that can't easily avoid the storm track [65,66]. It has been suggested that such storms starve seabirds, with their inability to feed cited as a cause of starvation [67]. A sharp temporary increase in turbidity brought about by intensified wave action and seabed shear stress may contribute to this incapacity, compounded by reduced ambient light levels and turbulence in the upper water column that accompany storms. Similarly, climate change is altering the location, timing, and intensity of planktonic blooms, which can severely limit visibility for months at a time over vast areas [68]. Anomalous blooms occurring in important seabird habitat have resulted in mass die-offs due to starvation [33], with associated turbidity likely to compromise seabirds' ability to locate prey. Increased turbidity has also been linked with elevated bycatch rates of seals by static gillnet fisheries [44]. Though this connection has not been investigated in other marine predators or fisheries, turbidity could contribute to bycatch risk for other species, and this topic deserves further attention.

Turbidity is currently overlooked as a dynamic descriptor of oceanic habitats, despite its potential to constrain the foraging abilities of many marine species. A changing climate brings with it altered physical attributes of ocean habitats including pH, temperature and optical properties of seawater. Understanding species’ sensory perception is vital to understanding how they function and their sensitivity to change, and as biologging technology continues to improve, we can improve our understanding of sensory cues that animals use to navigate and forage.

## Data Availability

All data and R scripts that were used to run this analysis are available at https://github.com/JamieHDarby/manxie_turbidity. Electronic supplementary material is available online [69].
